# Notes and Notices

**Published:** 1893-07

**Authors:** 


### NOTES AND NOTICES.

Chicago Baptist Hospital.—During the summer of 1891, Dr. L. D. Rogers with the assistance of a few friends organized and established the Chicago Baptist Hospital. On the 27th of last September, the institution celebrated its first anniversary. The ladies from thirty-three Baptist churches in and about Chicago gave a reception at the hospital and served refreshments to more than a thousand guests, among whom were many of the elite of the city.

The Chicago *Evening Post* considered the reception a society event of such importance as to give it a two-column report. The Chicago Baptist Hospital is the only denominational hospital in Chicago that has a homœopathic staff. It is also the largest homœopathic hospital in Chicago, and on account of its large and influential constituency it will probably always remain the largest. It is the only hospital in Chicago open alike to all homœopathic physicians.

